# A transcriptomic analysis of the effect of genistein on *Sinorhizobium fredii* HH103 reveals novel rhizobial genes putatively involved in symbiosis

**DOI:** 10.1038/srep31592

**Published:** 2016-08-19

**Authors:** F. Pérez-Montaño, I. Jiménez-Guerrero, S. Acosta-Jurado, P. Navarro-Gómez, F. J. Ollero, J. E. Ruiz-Sainz, F. J. López-Baena, J. M. Vinardell

**Affiliations:** 1 Departamento de Microbiología, Facultad de Biología, Universidad de Sevilla. Sevilla, Spain

## Abstract

*Sinorhizobium fredii* HH103 is a rhizobial soybean symbiont that exhibits an extremely broad host-range. Flavonoids exuded by legume roots induce the expression of rhizobial symbiotic genes and activate the bacterial protein NodD, which binds to regulatory DNA sequences called *nod* boxes (NB). NB drive the expression of genes involved in the production of molecular signals (Nod factors) as well as the transcription of *ttsI*, whose encoded product binds to *tts* boxes (TB), inducing the secretion of proteins (effectors) through the type 3 secretion system (T3SS). In this work, a *S. fredii* HH103 global gene expression analysis in the presence of the flavonoid genistein was carried out, revealing a complex regulatory network. Three groups of genes differentially expressed were identified: i) genes controlled by NB, ii) genes regulated by TB, and iii) genes not preceded by a NB or a TB. Interestingly, we have found differentially expressed genes not previously studied in rhizobia, being some of them not related to Nod factors or the T3SS. Future characterization of these putative symbiotic-related genes could shed light on the understanding of the complex molecular dialogue established between rhizobia and legumes.

The symbiotic relationship between legumes and nitrogen-fixing rhizobia involves reciprocal communication by means of chemical signals produced by the plant and the bacterium. Plant root secreted flavonoids are phenolic compounds that act as inducers of the bacterial *nod* genes, which encode enzymes for the production of specific lipochitooligosaccharidic signal molecules or Nod factors (NFs) that, in turn, are responsible for root infection and induction of nodule development. Within this new organ, differentiated bacteria reduce atmospheric nitrogen to ammonia, which is assimilated by the host plant in exchange of a carbon source and an appropriate environment that promotes bacterial growth^1^,^2^.

The regulatory protein NodD is constitutively expressed and codes for a LysR-type transcriptional activator that, in the presence of specific flavonoids, recognizes and binds to *nod* boxes (NB), promoter sequences located upstream of nodulation genes, triggering their transcription^3^,^4^,^5^. Interestingly, many other symbiotic-related traits such as polysaccharide production, phytohormone synthesis, motility, quorum sensing, and the activation of the type 3 secretion system (T3SS) are regulated, depending on the rhizobial strain, by specific inducer flavonoids and NodD^1^,^6^,^7^,^8^,^9^,^10^,^11^. However, to our knowledge, only a few genome-wide transcriptomic analyses of the effect of flavonoids on rhizobial gene expression have been performed so far. In *Bradyrhizobium japonicum* USDA110 about 100 genes were induced with genistein, including all *nod* box-associated genes, type 3 secreted effectors, the flagellar cluster, and several genes likely involved in transport processes^12^. Recently Huyghe *et al*.^13^ reported that many *Sinorhizobium fredii* NGR234 genes responded to the presence of daidzein: a total of 754 genes with a cutoff [fold-change value] ≥ 2. Although *nod* box- and T3SS-associated genes were those showing the highest levels of induction, these results are in agreement with those reported for *B. japonicum*, that indicate that flavonoids have a much broader function than the mere induction of the *nod* genes.

*Sinorhizobium fredii* nodulates more than a hundred genera of legumes, including plants forming determinate and indeterminate nodules such as *Glycine max* and *Glycyrrhiza uralensis*, respectively^14^,^15^,^16^. There is wide genomic information of the three most studied *S. fredii* strains: NGR234, USDA257, and HH103^17^,^18^,^19^,^20^. The HH103 genome is composed of 7 different replicons which harbour 6960 coding sequences (CDSs)^20^: the chromosome (4,305,723-bp, 4014 CDSs) and 6 plasmids: pSfHH103e/plasmid e (2,096,125-bp, 1991 CDSs), pSfHH103d/plasmid d/symbiotic plasmid (pSym, 588,797-bp, 667 CDSs), pSfHH103c/plasmid c (144,082-bp, 169 CDSs), pSfHH103b/plasmid b (61,880-bp, 62 CDSs), pSfHH103a1/plasmid a1 (24,036-bp, 19 CDSs) and pSfHH103a2/plasmid a2 (25,081-bp, 38 CDSs). HH103 harbours two copies of *nodD*, *nodD1* and *nodD2*, although only the former has been related to *nod* gene expression^10^. HH103 also possesses a copy of the *ttsI* gene, which codes for the T3SS transcriptional regulator TtsI^6^.

In 1999, Perret *et al*.^21^ defined 19 functional NB in the symbiotic plasmid of NGR234. Sequencing of the HH103 genome revealed that 4 of these NB (NB4, NB6, NB7, and NB11) are not present in this strain^20^. In addition, there are differences between NGR234 and HH103 in the genes driven by other 4 NB. Furthermore, 18 potential T3SS-promoter sequences or *tts* boxes (TB) were identified in the genome of HH103^20^. Many of the genes located downstream of all these regulatory sequences have not been studied in HH103 and some of them neither in other rhizobia.

In this work, RNA-seq and *q*RT-PCR (*q*PCR) studies were addressed to analyse the *S. fredii* HH103 global gene expression in the presence and absence of genistein, an effective *nod* gene inducer in this strain^10^. Three groups of genes differentially expressed upon treatment with genistein were identified: **i**) genes controlled by NB, **ii**) genes regulated by TB, and **iii**) genes not preceded by a NB or a TB. Consequently, further studies were performed with mutants affected in the regulatory genes *nodD1* and *ttsI*. Thus, an expression map of the *S. fredii* HH103 whole genome in the presence of genistein is provided, with special emphasis on the role of NodD1 and TtsI in the regulation of genes whose expression was affected by the presence of this flavonoid.

## Results and Discussion

### Identification of the *Sinorhizobium fredii* HH103 genes differentially expressed upon induction with genistein.

In order to identify genes induced with genistein, RNA-seq libraries were generated from HH103 cultures grown in the presence and absence of this flavonoid. To determine the role of NodD1 and TtsI in the regulation of the expression of the HH103 genes affected by genistein, HH103 *nodD1* and *ttsI* mutant derivatives were also included in these studies. Two biological independent replicates were obtained and analyzed for each condition. Thus, 12 RNA-seq libraries were generated and sequenced, obtaining between 39 and 49 million reads in each condition, which indicates that similar amounts of data were generated in all cases. The general features of each run are shown in Supplementary Data 1. In addition, all samples showed more than 90% of properly pair reads. Three different RNA-seq metrics for quality control, GC content, duplicate distribution, and the distribution of respective genetic coordinates, were performed (Supplementary Data 1). A normalization of the quantification data was also needed before all subsequent analysis to avoid statistical deviations due to differences in library and genetic sizes^22^ (Supplementary Data 1).

**Figure 1:**
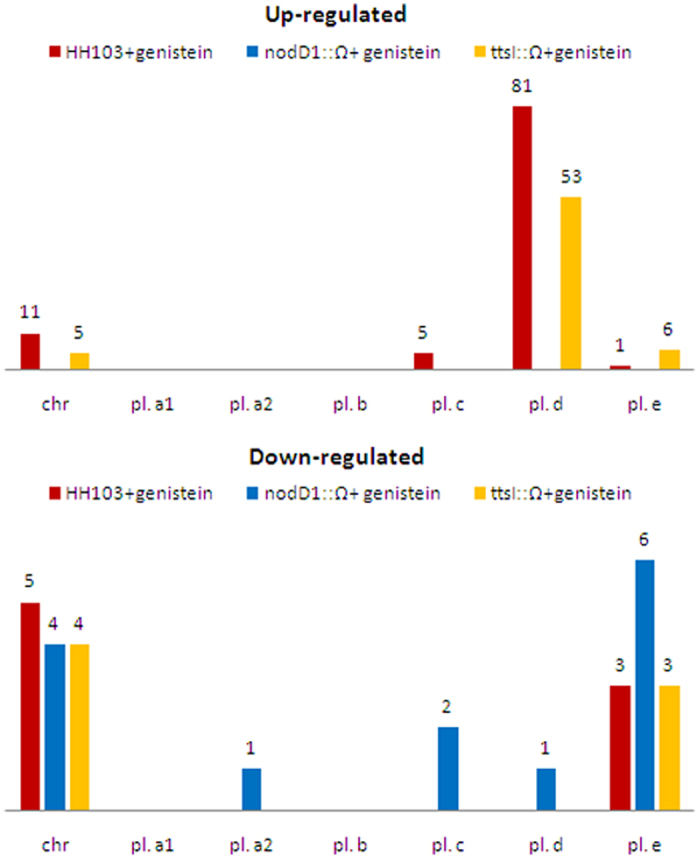
Replicon distribution and number of differentially expressed genes in *S. fredii* HH103 upon induction with genistein. Chr: chromosome, pl. a1: pSfHH103a1, pl. a2: pSfHH103a2, pl. b: pSfHH103b, pl. c: pSfHH103c, pl. d: pSfHH103d, and pl. e: pSfHH103e. Red bars: wild-type strain, blue bars: *nodD1* mutant; yellow bars: *ttsI* mutant.

Differentially expressed genes (DEG) in each strain upon treatment with genistein in comparison to the wild-type strain grown in the absence of genistein were detected as described in Methods. Our first criterion was the selection of DEG showing [fold-changes] ≥3 (i.e. [log_2_] >1.6). Thus, 106 (1.52% of the genome), 14 (0.2%), and 71 (1.02%) DEG were detected in the wild-type, *nodD1* and *ttsI* mutant strains, respectively, when compared to the wild-type strain grown in the absence of flavonoids (Fig. 1; Supplementary Data 2). The transcription of 18 of these genes was quantified by *q*PCR to validate the data set (Supplementary Table S1). In 17 out of these 18 genes, a linear correlation was obtained in the fold-change values obtained by both *q*PCR and RNA-seq (Supplementary Data 3).

In the wild-type strain, 98 genes were up-regulated in the presence of genistein and most of them (81) were located on the pSym. The rest of activated genes were found in the chromosome (11) and in plasmids c (5) and e (1). Only 8 genes were down-regulated in this condition (5 in the chromosome and 3 in plasmid e). Table 1 provides the list of the *S. fredii* HH103 genes showing the highest variations in expression (log_2_ fold-changes >3.3 or <−2) upon genistein treatment. The entire list of 106 DEG can be found as Supplementary Data 2. In addition to those related to the symbiotic T3SS and to the production of Nod factors, a large number of the genes over-expressed in these conditions coded for hypothetical proteins. These results indicate that a significant part of the genistein-induced regulon remains to be characterized.

The 14 DEG found in the *nodD1* mutant in the presence of genistein were all down-regulated and distributed among the chromosome (4) and plasmids e (6), c (2), d (1), and a2 (1). In the *ttsI* mutant, 64 out of the 71 differentially expressed genes were up-regulated, and most of them (53) were located on the symbiotic plasmid. The rest of the activated genes were found in plasmid c (6) and the chromosome (5). Down-regulated genes were located on the chromosome (4) and on plasmid e (3) (Fig. 1).

**Figure 2:**
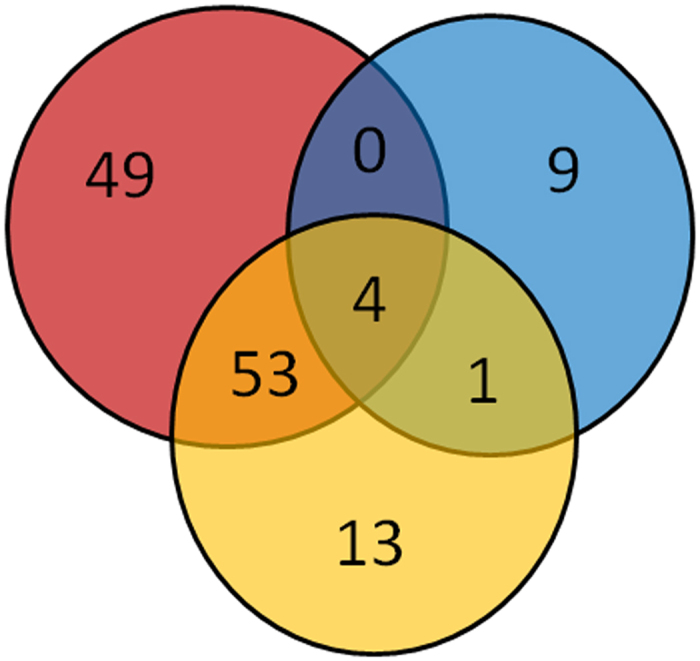
Comparative analysis of the set of genes regulated by genistein in *S. fredii* HH103 (red circle), and its *nodD1* (blue circle) and *ttsI* (yellow circle) mutant derivatives. The differences among these strains are visualized by a Venn diagram. The number of genes that are either individual for a certain strain or that are affected in two or three strains are indicated.

Figure 2 shows a comparative analysis of the sets of DEG upon treatment with genistein in HH103 and its *nodD1* and *ttsI* derivatives. The 53 genes shared by HH103 and its *ttsI* mutant derivative are those regulated via NodD1 (directly or indirectly), with the exception of those genes regulated by both NodD1 and TtsI, which should be part of the 49 DEG identified only in the wild-type strain. We also found 4 genes that are affected by genistein in the three strains, indicating that they were regulated in a NodD1- and TtsI-independent manner.

Interestingly, we found a gene, SFHH103_00387 (annotated as *fbpA*), which was clearly repressed in the *nodD1* and *ttsI* mutant backgrounds regardless the presence or absence of genistein. This pattern of expression was confirmed by *q*PCR (Supplementary Table S1, Supplementary Data 3). The product of this gene is predicted to be a periplasmic protein involved in the uptake of Fe^3+^. Whether this *S. fredii* HH103 protein is involved in Fe uptake and/or the reason why its expression is repressed in the absence of NodD1 or TtsI requires further investigation.

### The *S. fredii* HH103 genes affected by genistein can be assigned to three different groups based on their promoter sequences

As mentioned above, RNA-seq analyses led to the identification of 106 *S. fredii* HH103 DEG upon treatment with genistein. However, we also considered genes that, showing lower changes of expression (as *noeI* or *nolK*), belonged to operons in which some of the genes exhibited clear genistein-mediated differential expression. The two copies of *nopM* present in the HH103 genome (expression fold-changes of 2.1 and 2.2) were also included because they were preceded by a well-conserved TB. Thus, the number of genes considered as affected by genistein in *S. fredii* HH103 raised to 117. Sixteen out of these 117 genes, however, were discarded by different reasons such as showing opposite direction to known *nod* boxes or being identical copies of a gene under the control of a NB (Supplementary Data 4). Therefore, only 101 genes, which could be assigned to three different groups based on the presence or absence of functional NB or TB in their promoter sequences, were subjected to further analyses.

### *S. fredii* HH103 genes induced through *nod* boxes (NB)

Flavonoids exuded by legume roots activate the transcription of *nod* genes through their interaction with NodD. Although this protein interacts with specific promoter sequences (NB) even in the absence of inducers, binding of an appropriate flavonoid to NodD enhances the access of the RNA polymerase and increases the transcriptional level of the *nod* genes^5^,^23^.

The present study led to the functional study of the 15 NB present in the HH103 symbiotic plasmid^20^. Those NB located upstream of genes induced with genistein (>3 fold) in the wild-type and the *ttsI* mutant strains, but not in the *nodD1* mutant, were considered active: NB1, NB2, NB8, NB9, NB10, NB13, NB14, NB15, NB17, NB18, NB19 (Supplementary Table S2, Supplementary Data 4). The genistein-induced expression of genes belonging to NB1, NB8, NB10, NB13, NB14, NB15, NB17 and NB19 has been validated by *q*PCR in this work (Supplementary Table S1) and that of *noeL* (NB2) and *ttsI* (NB18) has been previously demonstrated^6^,^24^. Quantitative PCR experiments confirmed that expression of psfHH103d_118 (NB9) was inducible by genistein in a NodD1-dependent manner (8.2-fold). Once the active NB were determined, 36 ORF located downstream these promoters were identified (Supplementary Table S2, Supplementary Data 4).

NB3 is situated upstream of *nodD1* but opposed to this gene. In *S. fredii*, in contrast to other rhizobia, *nodD1* is not located close to the common *nodABC* genes. Therefore, NB3 might be a reminiscence of a promoter controlling the transcription of *nodABC* in a *S. fredii* ancestor. The gene whose expression is controlled by NB3 (psfHH103d_384) codes for a hypothetical protein not present in other rhizobia, and was induced 2.5-fold in the presence of genistein in HH103 and in its *ttsI* mutant, but not in the *nodD1* derivative, suggesting that NB3 is active but shows a low efficiency.

*S. fredii* HH103 NB5 and NB16 lack downstream genes^20^. The downstream DNA regions showed very low number of reads regardless the presence of flavonoids and/or NodD1 (data not shown), which indicates that both NB are not functional. In addition, the absence of induction by genistein of the pseudogenes *nodS* and *nodU* of *S. fredii* HH103 indicated that the preceding NB12 was either not functional. These results are in agreement with those obtained in USDA257^25^ but in contrast to the situation described in NGR234^21^.

**Figure 3:**
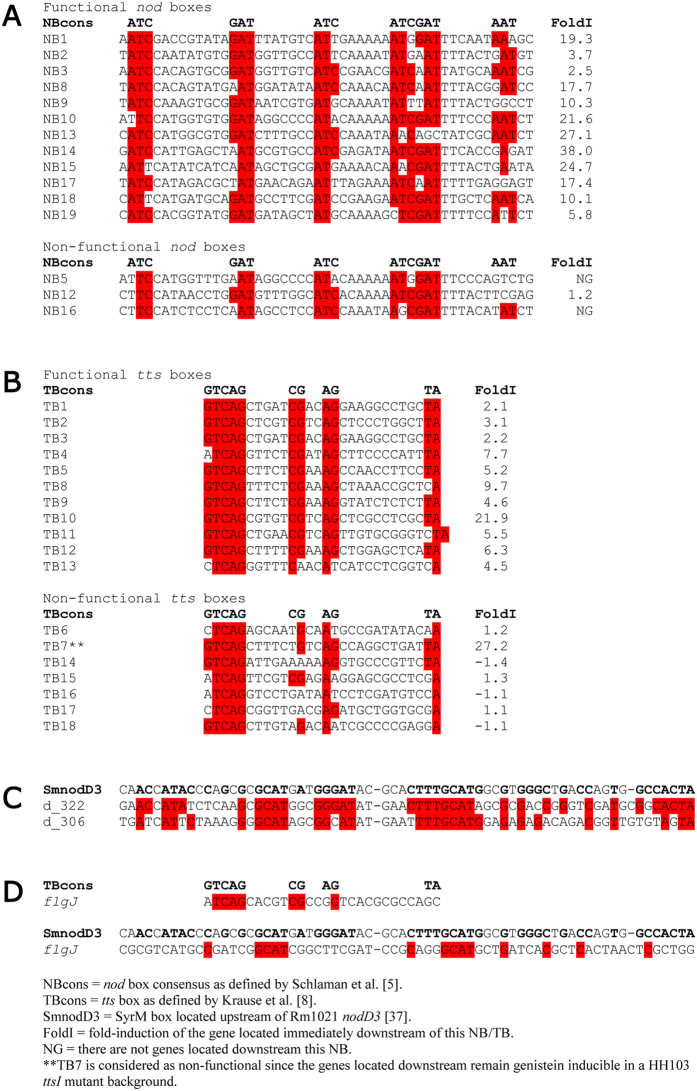
Identified promoter sequences driving genistein-mediated expression in *S. fredii* HH103. The fold-induction with genistein of the gene located immediately downstream is shown. (**A**) functional and non-functional *nod* boxes. (**B**) functional and non-functional *tts* boxes. (**C**) putative SyrM boxes located upstream of psfHH103d_306 and psfHH103d_322. (**D**) Imperfect *tts* and SyrM boxes located upstream of SFHH103_00346 (*flgJ*).

Figure 3A shows the alignments of the functional and non-functional NB of *S. fredii* HH103. Curiously, none of them, regardless their transcriptional activity, entirely follow the consensus NB sequence defined by Schlaman *et al*.^3^: ATCN_9_GATN_7_ATCN_6_ATCGATN_6_AAT. The three non-functional NB lack the initial A residue and fail in the last three nucleotides motif (AAT), suggesting that these residues could be very relevant for the functionality of this promoter region. Although the functional NB10 also fails in the initial A residue, it does contain the final AAT motif. We could not find a clear correlation between the sequences of the HH103 functional NB and their strength (fold induction of the gene located immediately downstream). Clearly, further research is required to clarify this issue.

Some of the *S. fredii* HH103 functional NB control the expression of genes previously related to symbiosis (Supplementary Table S2, Supplementary Data 4). NB2 and NB8 are responsible for the expression of *nodZnoeLnolK* and *nodABCIJnolO’noeI*, respectively, which are dedicated to the synthesis and export of the different HH103 Nod factors^20^. NB14 controls the expression of the *fixABCX* genes, conserved in rhizobia and essential for nitrogen fixation in the *S. meliloti*-alfalfa symbiosis, since they probably code for a putative membrane complex participating in electron transfer to nitrogenase^26^,^27^. NB15 drives the expression of psfHH103d_257, which is involved in the flavonoid-induced synthesis of indole acetic acid (IAA)^20^, a phytohormone related to plant root growth promotion. Flavonoid-induced production of IAA has also been reported for *S. fredii* NGR234 and *Rhizobium tropici* CIAT 899^9^,^28^, suggesting that promotion of legume root growth could be widespread among rhizobia.

NB1 controls the expression of 5 genes (psfHH103d_373 to psfHH103d_370) possibly related to the synthesis of pentacyclic triterpenoids called hopanoids. It has been reported very recently that inactivation of the orthologous genes of *Bradyrhizobium* sp. and *Bradyrhizobium diazoefficiens*, whose expression is not driven by NB, negatively affects bacterial survival in stressful conditions and symbiotic performance with their host legumes^29^,^30^. The fact that in *S. fredii* HH103 hopanoid-related genes are induced by flavonoids in a NodD1 dependent manner suggests that hopanoids could have a bacterial protective role during symbiosis, although further research is required to elucidate this point.

On the other hand, RNA-seq analysis indicated that four NB (NB9, NB10, NB13, and NB17) control the expression of genes coding for conserved hypothetical proteins whose functions are currently unknown or poorly investigated (Supplementary Table S2, Supplementary Data 4). Future efforts must be focused on the study of these NodD1-flavonoid-activated genes, since they could shed light on molecular aspects of the symbiotic interaction between *S. fredii* HH103 and its legume hosts. As an example, *S. fredii* HH103 exopolysaccharide (EPS) production is inhibited by genistein^11^. However, among the DEG found upon a 24 h genistein treatment, there were not known genes (*exo*/*exs*) coding for enzymes directly involved in EPS production. Regarding EPS regulatory genes, in *S. meliloti* at least 8 proteins control succinoglucan (EPS I) production [reviewed by^31^]: MucR and SyrM act as positive regulators, whereas ExoX, ExoR, ExoS, ExsB, CbrA and EmmC repress this process. In *S. fredii* HH103, with the exception of *syrM* (fold-change +5.8, see below), the expression of the orthologous genes of these regulators was not greatly affected by the presence of genistein (fold-changes of −1.1 to +1.9). In addition to the putative involvement of *syrM* in the process, there is another HH103 gene, psfHH103d_161, that could be related to regulation of EPS production. This gene, which is under the control of NB10, is highly induced by genistein (fold-change +21.6) and codes for a hypothetical protein 58% identical to the *R. leguminosarum* biovar *phaseoli* PsiB. The fact that PsiB is involved in exopolysaccharide inhibition^32^ opens the possibility that the product encoded by psfHH103d_161 could be related to the genistein-mediated EPS repression exhibited by HH103.

The expression of two genes coding for transcriptional regulators, *ttsI* (NB18) and *syrM* (NB19), was also up-regulated via NodD1 and genistein (Supplementary Table S2, Supplementary Data 4). TtsI is the main activator of the *S. fredii* HH103 T3SS^6^ and SyrM is known to be a transcriptional regulator implied in the optimal performance of the *S. meliloti*-*Medicago* symbiosis^33^. This finding indicates that in *S. fredii* HH103, in addition to the expression of the symbiotic T3SS through TtsI, NodD1 and inducer flavonoids could activate the expression of a set of genes via SyrM. Moreover, NB19 controls the genistein-induced expression of two other genes: psfHH103d_368 and psfHH103d_369, which code for a putative transcriptional regulator, and a 114-residue hypothetical protein, respectively.

### *S. fredii* HH103 genes induced through *tts* boxes (TB)

Some Gram-negative bacterial strains possess a specialized apparatus for protein secretion termed T3SS. Pathogenic and symbiotic bacteria, including rhizobia, use this system to deliver effector proteins directly into the eukaryotic host cell^34^ in order to interfere with the host signal transduction cascades and promote pathogen or symbiotic infection by suppressing host defenses^35^,^36^. In rhizobia, these proteins are called Nops (Nodulation outer proteins).

*S. fredii* strains NGR234 and HH103 possess a T3SS that is induced by flavonoids and depends on NodD1 since expression of *ttsI*, the T3SS transcriptional regulator, is driven by a NB^6^. Thus, up-regulation of all T3SS genes is mediated by TtsI through binding to specific promoter sequences called *tts* boxes (TB). As previously mentioned, HH103 harbors 18 potential TB distributed among several replicons^20^. Our present study allowed the functional verification of these potential promoter regions, considering active those TB located upstream of genes transcriptionally activated with genistein in the wild-type strain but not in the *nodD1* and *ttsI* mutants. RNA-seq analysis showed that only 11 *tts* boxes (TB1, 2, 3, 4, 5, 8, 9, 10, 11, 12, and 13) were active, controlling the expression of 35 ORFs (Supplementary Table S2, Supplementary Data 4). Although the genes located downstream of TB7 (psfHH103d_322-to psfHH103d_319) showed enhanced expression in the presence of genistein, this *tts* box was considered non-functional since this pattern of induction was not altered by the inactivation of *ttsI*. As discussed later, the upstream sequence of this genetic region (which includes the transcriptional regulator *nodD2*) contains a putative SyrM box which might explain why these genes are induced by genistein.

Most of the HH103 functional TB were located on the symbiotic plasmid, with the exception of TB1 and TB2, which were present in plasmid c. The differential expression of *gunA* (TB5), *nopL* (TB9), *nopC* (TB12), and *nopI* (TB2) was validated by *q*PCR (Supplementary Table S1; Supplementary Data 3) and previous works have shown that the expression of *rhcJ* (TB10), *nopA* (TB12), *nopP* (TB11), and *nopX* (TB8) was also regulated by flavonoids, NodD1 and TtsI^6^,^37^. All functional TB mainly followed the consensus GTCAGN_5_CGN_2_AGN_10_TA while the non-active TB failed to maintain the internal sequence CGN_2_AG (Fig. 3B). Four of these active TB (TB5, 8, 10, and 12) control the expression of operons coding for proteins that i) are components of the T3SS apparatus (such as *nopBrhcJnolUrhcLNQRSTU* and *nopCA*y4yQ, which depend on TB10 and TB12, respectively), ii) are implied in the translocation of proteins to the cytoplasm of the root cell (the *nopX* operon controlled by TB8), or iii) code for glycolitic enzymes (the *gunA* gene controlled by TB5) (Supplementary Table S3, Supplementary Data 4). The rest of the active *tts* promoter sequences (TB1, 2, 3, 4, 9, 11, and 13) drive the expression of genes that code for the putative effector proteins NopI, NopM/M2, NopD, NopL, NopP and NopT. These results are in agreement with previous reports in which the type 3-dependent secretion of at least nine *S. fredii* HH103 Nops (NopA, NopB, NopC, NopD, NopL, NopM/NopM2, NopP, and NopX) has been demonstrated^6^,^38^. NopA, NopB, and NopX are the main components of the T3SS extracellular appendages^39^,^40^,^41^. Secretion of NopP and NopC to the interior of *Vigna unguiculata* and *Glycine max* nodule cells, respectively, has been recently confirmed^42^,^43^ and the rest can be considered putative effectors. Results shown in this work allowed the identification, for the first time in a rhizobial strain, of the complete repertoire of T3SS-effectors. However, we cannot discard the future identification of other effectors that do not follow the up to date accepted signaling cascade that involves flavonoids, NodD1 and TtsI.

The confirmation that *gunA* and *nopI* were transcriptionally activated in a flavonoid-, NodD1-, and TtsI-dependent manner was another interesting finding. GunA, previously identified only in *B. japonicum*, is a cellulase that could be implied on the breaking of the plant cell wall to insert the inyectisome in the plant cell membrane and allow protein translocation directly into the eukaryotic cytoplasm^44^. NopI, located on plasmid c downstream TB2, could be a new type of rhizobium-specific T3SS effector not previously described^20^ but further efforts are necessary to identify unequivocally this protein as a HH103 secreted effector. Interestingly, *nopI* appears to be the first gene of an operon that also contains three genes (SFHH103_04165 to SFHH103_04167) encoding the N-terminal, middle and C-terminal parts of a putative reverse transcriptase whose functionality and putative symbiotic involvement remain to be investigated.

### *S. fredii* HH103 genes not controlled by NB or TB but regulated by genistein

The last set of HH103 DEG in the presence of genistein corresponds to those lacking defined NB or TB promoter regions. Our study revealed that 30 *S. fredii* HH103 ORFs can be included in this group (Supplementary Table S3, Supplementary Data 4). These genes were distributed among the chromosome and plasmids d and e, being 22 of them up-regulated and 8 down-regulated in the presence of genistein. The absence of known NB or TB promoter regions does not necessarily imply transcriptional independence of NodD1 and TtsI. In fact, the genistein-mediated differential expression of 24 of these 30 genes depends on the presence of a functional NodD1 protein, being 7 out of these 24 genes apparently also TtsI-dependent (Supplementary Table S3, Supplementary Data 4). This finding indicates that *S. fredii* HH103 NodD1 regulates a set of genes that do not depend on NB or TB. Regarding genes depending on NodD1, it could be possible that this additional level of regulation was mediated, at least partially, by the NB-controlled transcriptional regulator SyrM (NB19, see above). In this sense, well conserved SyrM boxes, as defined by Barnett and Long in the closely related *S. meliloti* Rm1021^33^, were found upstream two operons that were highly induced by genistein and lacked functional NB or TB (Fig. 3C, Supplementary Table S3, Supplementary Data 4): psfHH103d_322-psfHH103d_319 (encoding hypothetical proteins and the regulatory protein NodD2) and psfHH103d_306-psfHH103d_311 (encoding hypothetical proteins putatively involved in electron transfer). As mentioned above, the *tts* box located upstream of psfHH103d_322- psfHH103d_319 has been considered as non-functional because this operon remains genistein-inducible in a *ttsI* mutant background. Putative SyrM boxes were located at positions −223 to −158 and −127 to −62 upstream the translation initial site of psfHH103d_322 and psfHH103d_306 respectively, but its functionality remains to be demonstrated.

The genistein-NodD1-induced expression of psfHH103d_306 was confirmed by *q*PCR (Supplementary Table S1, Supplementary Data 3). *q*PCR experiments also showed that HH103 *nodD2* is positively regulated by NodD1 and genistein (±6.1 ± 1.1), suggesting that in *S. fredii* HH103 the expression of *nodD2* (and the other genes of the operon) was most probably regulated through SyrM, as previously reported for the closely related strain NGR234^45^. Thus, in *S. fredii* HH103, regulation of symbiotic genes by flavonoids appeared to be rather complex since it involved NodD1, TtsI, NolR and possibly SyrM and NodD2. In fact, the latter has been reported to act as a repressor of *nodD1* expression in *S. fredii* NGR234 and USDA191^45^,^46^. Further transcriptomic analyses of the role of NodD2, SyrM and NolR in gene regulation are required to determine the general regulatory circuit that controls the most basic aspects of the molecular dialogue involved in the symbiotic interaction between HH103 and its host legumes.

We have found several genes that are inducible by genistein and NodD1 and that do not contain NB, TB or SyrM boxes in their upstream regions, which indicates that unknown actors mediating the flavonoid-NodD1 regulatory cascade remain to be discovered. Among these genes, the expression pattern of psfHH103d_255 and SFHH103_02192 has been confirmed by *q*PCR (Supplementary Table S1, Supplementary Data 4). psfHH103d_255 is located on plasmid d and codes for a putative membrane protein belonging to the major facilitator superfamily MFS-1. SFHH103_02192 is situated in the chromosome and its predicted product is a hypothetical protein containing a complete COG2931 domain (Ca^2+^-binding protein, RTX toxin-related) as well as two incomplete Peptidase_M10_C domains (Peptidase M10 serralysin C terminal). Another chromosomal gene induced by NodD1 and genistein but not controlled by known regulatory promoters was *ligE* (SFHH103_00841), which codes for a β-aryl ether cleaving enzyme containing a GstA (gluthatione S-transferase, GST) domain. This kind of proteins is usually implied in detoxification processes. Plant GSTs are abundant in nodules and likely function to provide antioxidant defenses that are critical to support nitrogen fixation^47^. To our knowledge, this is the first time that a rhizobial (putative) GST is found to be inducible by NodD1 and flavonoids.

As mentioned above, our results indicated that the differential expression upon treatment with genistein of 7 CDSs with unknown promoter regions depended on the presence of both NodD1 and TtsI. Among these genes, we found a putative operon located on the chromosome that is composed of *flgJ* and two genes coding for conserved hypothetical proteins (Supplementary Table S3, Supplementary Data 4). The expression pattern of *flgJ* was confirmed by *q*PCR (Supplementary Table S1). *flgJ* codes for a flagellar protein containing a scaffolding domain required for polymerization of the distal rod of the flagellum^48^. Analysis of the upstream sequence of *flgJ* revealed the presence of imperfect *tts* and SyrM boxes (Fig. 3D), located at positions −152 to −126 and −817 to −752 upstream the translation initial site of *flgJ*, but to elucidate whether the differential expression of this operon depends on these putative promoter sequences requires further investigation. Nevertheless, the fact that *flgJ* was induced by genistein opens the possibility that flavonoids could affect HH103 motility. We plan to investigate this hypothesis in the next future. Interestingly, the bacterial *flagellum* shares a common ancestor with the T3SS^49^, which suggests that up-regulation of this gene could be related with the correct formation and assembly of T3SS machinery across the bacterial cell wall.

Finally, RNA-seq analysis showed that 4 genes were affected by genistein independently of the presence of NodD1 or TtsI. One of these genes, SFHH103_03875, located on the chromosome and induced with genistein (~6-fold), codes for a putative membrane permease containing two EamA domains. Database searching showed that this gene is well conserved among different rhizobia, but to our knowledge it has not been characterized. In addition, a set of three genes situated on plasmid e (SFHH103_05321 to SFHH103_05319) was repressed by the presence of genistein independently of NodD1 and TtsI, as validated by *q*PCR (Supplementary Table S1). This operon codes for conserved hypothetical proteins showing similarities with a TetR transcriptional regulator and two multidrug efflux transporter proteins (Supplementary Data 4). Recently, Rossbach and coworkers^50^ showed that three genes coding for a TetR regulator (EmrR) and two components (EmrAB) of an efflux system were induced by flavonoids in *S. meliloti*, most probably in a NodD-independent way. In addition to the different effect of flavonoids on their expression, *emrAB* are not orthologues of SFHH103_05320 and 05319. Thus, it is intriguing why two closely-related rhizobia harbor different efflux systems regulated by the presence of flavonoids but independently of NodD and why the efflux system of *S. meliloti* is induced whereas that of *S. fredii* is repressed.

## Conclusion

**Figure 4:**
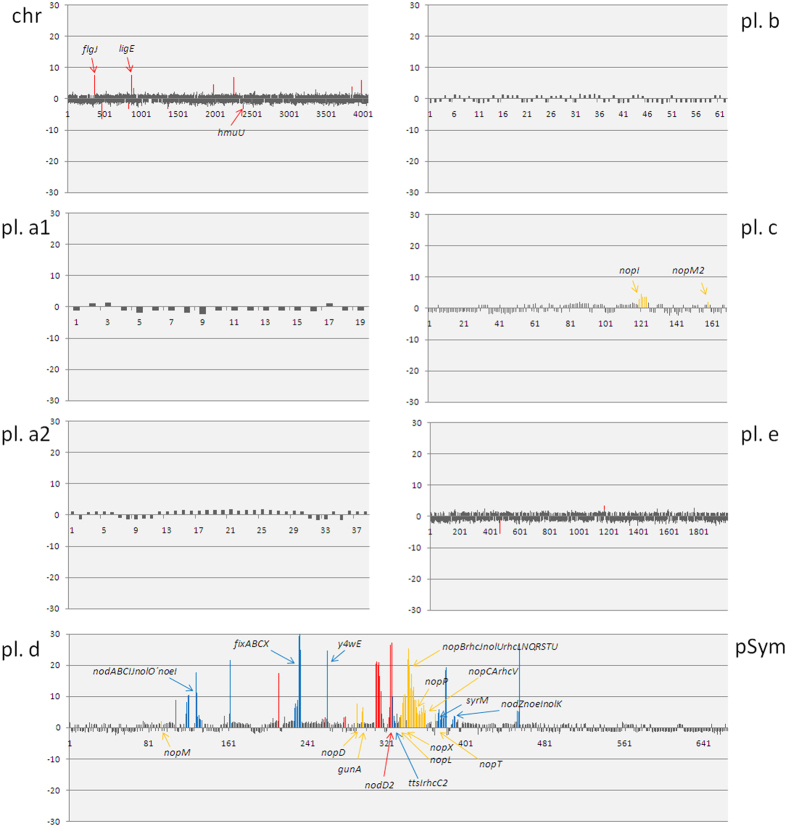
Linear representation of the complete RNA-seq-based transcriptomic data set of bacterial cultures induced with genistein for all replicons of *S. fredii* HH103. Bars represent fold-change expression values. Each bar corresponds to one gene, being ordered according to their relative position in the replicon. Chr: Chromosome, pl. a1: pSfHH103a1, pl. a2: pSfHH103a2, pl. b: pSfHH103b, pl. c: pSfHH103c, pl. d (pSym): pSfHH103d and pl. e: pSfHH103e. Blue bars: ORFs regulated by genistein preceded by *nod* boxes and regulated by NodD1. Yellow bars: ORFs regulated by genistein preceded by *tts* boxes and regulated by TtsI. Red bars: ORFs regulated by genistein but not dependent on *nod* or *tts* boxes. Gene names shown correspond to those genes that have been annotated.

**Figure 5:**
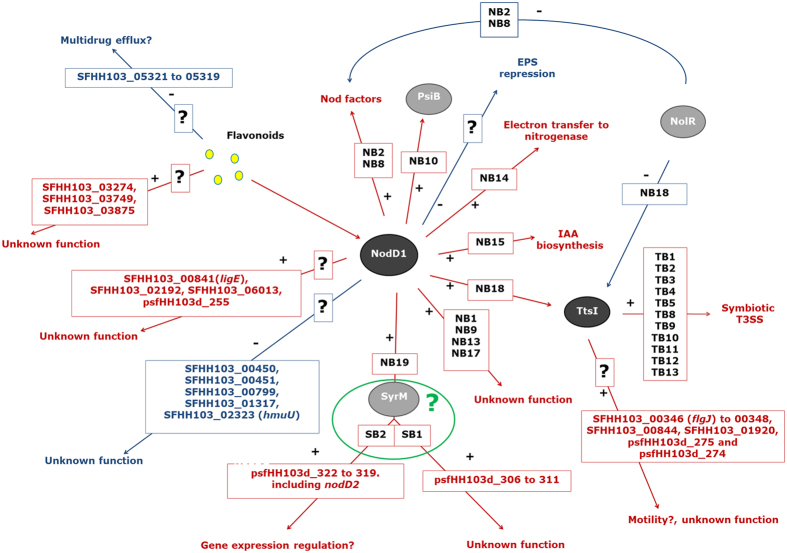
Model of the genistein stimulon of *S. fredii* HH103. This scheme summarizes the information obtained in this work as well as results about the NolR repressor protein previously reported^11^. Induction and repression of genes (and processes) are indicated in red and in blue arrows, respectively. NB, *nod* box; TB, *tts* box; SB, putative SyrM box. Framed question marks indicate the possibility of the participation of unknown regulatory elements. The green circle surrounding SyrM, SB1 and SB2 denotes the putative involvement of these elements in the NodD1-genistein-mediated induction of psfHH103d_306-311 and psfHH103d_322-319.

A better understanding of the molecular mechanisms operating in the rhizobia-legume symbiosis is a requisite for the improvement of this interaction as well as for its putative extension to other families of plants of ecological and agronomical importance. The RNA-seq and *q*PCR analyses shown in this work define three groups of genes differentially expressed upon induction with genistein on *S. fredii* HH103: genes controlled by NB (e.g. *nod* and *fix* regions, *ttsI* and *syrM* genes), genes regulated by TB (e.g. *rhc* and *nop* regions) and genes not preceded by a NB or a TB (e.g. *ligE* and *flgJ* genes) (Fig. 4). A scheme summarizing the genistein stimulon (i.e., the set of genes whose expressions is affected by the presence of this flavonoid) of *S. fredii* HH103 is shown in Fig. 5. As can be observed, various regulatory elements remain to be identified. Our results are in agreement with previous reports indicating that *nod* gene inducing flavonoids possess a much broader transcriptional effect than the mere induction of genes implied in production of Nod factors and Nops^12^,^13^. Thus, there are many other DEG that remain to be characterized. Further efforts will be needed to describe the function of these genes, which would improve our understanding of the molecular dialogue established between both symbionts.

## Methods

### Culture conditions and RNA extraction

Three *Sinorhizobium fredii* strains, namely HH103 Rif^R ^51, HH103 Rif^R^*nodD1*::*Ω*^10^ and HH103 Rif^R^*ttsI*::*Ω*^6^ were grown at 28 °C until stationary phase (OD_600_ ≈ 1,2) on yeast extract mannitol medium (YM)^52^, supplemented with genistein 3.7 μM when necessary. When required, the media were supplemented with the antibiotics rifampicin (50 μg ml^−1^) or spectinomycin (50 μg ml^−1^). Total RNA was isolated using a High Pure RNA Isolation Kit (Roche) according to the manufacturer’s instructions. Verification of the amount and quality of the resulting total RNA samples was carried out using a Nanodrop 1000 spectrophotometer (Thermo Scientific) and a Qubit 2.0 Fluorometer (Invitrogen). Two independent total RNA extractions were obtained for each condition.

### Quantitative reverse transcription PCR

Results obtained in the RNA-seq analysis were validated by quantitative reverse transcription PCR (*q*RT-PCR) of 19 selected genes, which represented differentially and non-differentially expressed genes in the three strains in the presence of genistein. Total RNA was isolated using a High Pure RNA Isolation Kit (Roche) and RNAase Free DNAse (Qiagen) according to the manufacturer’s instructions. This (DNA free) RNA was reverse transcribed to cDNA using a QuantiTec Reverse Transcription Kit (Qiagen). Quantitative PCR was performed using a LightCycler 480 (Roche) with the following conditions: 95 °C, 10 min; 95 °C, 30 s; 50 °C, 30 s; 72 °C, 20 s; forty cycles, followed by the melting curve profile from 60 to 95 °C to verify the specificity of the reaction. The *S. fredii* HH103 RNA *16S* gene was used as an internal control to normalize gene expression. The fold changes of two biological samples with three technical replicates in each condition were obtained using the ∆∆C_t_ method^53^. Selected genes and primers are listed in Supplementary Data 3.

### RNA sequencing

Ribosomal RNA was depleted using a MICROB Express Bacterial mRNA Purification kit (Ambion), following the manufacturer’s protocol. Integrity and quality of the ribosomal depleted RNA was checked with Agilent Bioanalyzer 2100 (Agilent Technologies). RNA sequencing was carried out by Sistemas Genómicos (https://www.sistemasgenomicos.com/web_sg/) with the Next Generation Sequence (NGS) platform Illumina using the Illumina HiSeq 2000 sequencing instrument (Illumina). Ribosomal-depleted samples were used to generate whole transcriptome libraries following the manufacturer’s recommendations for sequencing on this NGS platform. Amplified cDNA quality was analyzed by the Bioanalyzer 2100 DNA 1000 kit (Agilent Technologies) and quantified using the Qubit 2.0 Fluorometer (Invitrogen).

### Mapping of the RNA-seq data

The initial whole transcriptome paired-end reads obtained from sequencing were mapped against the latest version of the *S. fredii* HH103 genome (http://www.ncbi.nlm.nih.gov/assembly/GCF_000283895.1/) using the Life Technologies mapping algorithm version 1.3 (http://www.lifetechnologies.com/). Low-quality reads were eliminated using Picard Tools software version 1.83, remaining only high quality reads.

### Assessment of differentially expressed genes

Gene prediction was estimated using the cufflinks method^54^. Quantification of gene expression levels was evaluated using the htseq-count method software, version 0.5.4p3, and the algorithm proposed by DESeq2^55^,^56^. This method eliminates multimapped reads, considering only unique reads for the gene expression estimation. The edgeR method version 3.2.4 was applied for differential expression analysis among conditions^57^. This method uses a Poisson model to estimate the variance of the RNA-seq data for differential expressions, and relies on different normalized processes based on depth global samples, CG composition and length of genes. RNA-seq data of each treatment were compared to those of the wild-type strain grown in the absence of genistein. Differentially expressed genes were defined as those genes with a fold-change lower or higher than −3 or 3, respectively, with a *p* value inferior to 0.05.

### Consensus motifs

Functional *nod* and *tts* boxes of *S. fredii* HH103 identified by Vinardell *et al*.^20^ and validated by RNA-seq in this work were aligned using the ClustalW program and manipulated with Boxshade at EMBnet.

Search of conserved nucleotide motifs (such as SyrM boxes) was done by using the *fuzznunc* utility from the EMBOSS software package^58^.

Search of conserved protein motifs was performed at NCBI CD-search (http://www.ncbi.nlm.nih.gov/Structure/cdd/wrpsb.cgi).

### RNA-seq data accession number

The RNA-seq data discussed in this publication have been deposited in the Sequence Read Archive of NCBI (BioProject database) under the BioProject ID PRJNA313151.

## Additional Information

**How to cite this article**: Pérez-Montaño, F. *et al*. A transcriptomic analysis of the effect of flavonoids on *Sinorhizobium fredii* HH103 reveals novel rhizobial genes putatively involved in symbiosis. *Sci. Rep*. **6**, 31592; doi: 10.1038/srep31592 (2016).

## Supplementary Material

Supplementary Information

Supplementary Data 2

Supplementary Data 3

Supplementary Data 4

## Figures and Tables

**Table 1:** *S. fredii* HH103 genes showing the highest expression variations upon genistein treatment.

Gene Name	Gene ID	Log_2_ Fold-change	Description
—	psfHH103d_229	5.2	Putative iron-sulfur cluster assembly accessory protein
—	psfHH103d_228	4.9	Hypothetical protein
—	psfHH103d_322	4.8	Hypothetical protein
—	psfHH103d_448	4.8	Hypothetical protein
—	psfHH103d_321	4.7	Hypothetical protein
*rhcJ*	psfHH103d_338	4.7	Component RhcJ of symbiotic T3SS
—	psfHH103d_230	4.6	Hypothetical protein
—	psfHH103d_257	4.6	Histidinol-phosphate aminotransferase
*nopB*	psfHH103d_337	4.5	Nodulation outer protein NopB
—	psfHH103d_161	4.4	Conserved hypothetical protein
—	psfHH103d_307	4.4	Hypothetical protein
—	psfHH103d_309	4.4	Hypothetical protein
—	psfHH103d_306	4.4	Conserved hypothetical protein
—	psfHH103d_308	4.3	Conserved hypothetical protein
*nolU*	psfHH103d_339	4.3	Component NolU of symbiotic T3SS
—	psfHH103d_373	4.3	Hypothetical protein
—	psfHH103d_372	4.2	Squalene/phytoene synthase
*nodA*	psfHH103d_126	4.1	Acyl transferase (Nod factors synthesis)
—	psfHH103d_208	4.1	ABC-type transport system/periplasmic component
*rhcN*	psfHH103d_341	4.1	ATPase RhcN for symbiotic T3SS
—	psfHH103d_310	4.1	Hypothetical protein
*rhcQ*	psfHH103d_343	3.7	Component RhcQ of symbiotic T3SS
*rhcL*	psfHH103d_340	3.6	Component RhcL of symbiotic T3SS
—	psfHH103d_342	3.6	Conserved hypothetical protein
—	psfHH103d_311	3.6	Partial tryptophan halogenase
*nodB*	psfHH103d_127	3.5	Polysaccharide deacetylase (Nod factors synthesis)
—	psfHH103d_334	3.4	Hypothetical protein
—	psfHH103d_119	3.4	Hypothetical protein
—	psfHH103d_333	3.4	Conserved hypothetical protein
—	psfHH103d_118	3.4	Conserved hypothetical protein
*ttsI*	psfHH103d_323	3.3	TtsI, positive regulator of symbiotic T3SS
*nopX*	psfHH103d_335	3.3	Nodulation outer protein NopX
—	SFHH103_05320	−2.3	Multidrug efflux transporter protein
—	SFHH103_05321	−2.5	TetR family transcriptional regulator
—	SFHH103_00451	−2.7	Conserved hypothetical protein

Only genes showing log_2_ fold-changes >3.3 or <−2 are showed.
