# Supplementary material for: A transcriptomic analysis of the effect of genistein on Sinorhizobium fredii HH103 reveals novel rhizobial genes putatively involved in symbiosis
Source: Sci Rep. 2016 Aug 19;6:31592. doi: 10.1038/srep31592 (PMC4990936; doi:10.1038/srep31592)
Supplement: Supplementary Information [file srep31592-s1.pdf]

A transcriptomic analysis of the effect of genistein on *Sinorhizobium fredii* HH103 reveals novel rhizobial genes putatively involved in symbiosis.

Authors: Pérez-Montaño F, Jiménez-Guerrero I, Acosta-Jurado S, Navarro-Gómez P, Ollero FJ, Ruiz-Sainz JE, López-Baena FJ, Vinardell JM*.

Departamento de Microbiología, Facultad de Biología, Universidad de Sevilla. Sevilla, Spain.

Supplementary Information:

- Supplementary Tables

Table S1. RNA-seq data validation using *q*RT-PCR.

Table S2. 36 and 35 *Sinorhizobium fredii* HH103 ORFs are regulated by genistein through *nod* (NB) and *tts* (TB) boxes respectively.

Table S3. 30 *Sinorhizobium fredii* HH103 ORFs are regulated by genistein but are not dependent on *nod* or *tts* boxes.

- Supplementary Data 1. General features of the total sequenced and mapped reads.

- References cited in Supplementary Data 4.

Table S1. RNA-seq data validation using *q*RT-PCR (*q*PCR). The expression of 19 individual genes was measured in the wild-type strain and in its *nodD1* and *ttsI* mutant derivatives, both in the presence or absence of genistein. For *q*RT-PCR, fold-change values were calculated using the ΔΔCt method and normalized to the reference 16S rRNA gene.

**Gene name**	**Locus Tag / Promoter Sequences**	**RNA-seq**			***q*PCR**		
		**Wild-type**	**NodD1$^-$**	**TtsI$^-$**	**Wild-type**	**NodD1$^-$**	**TtsI$^-$**
-	psfHH103d_373 / NB1	19.3	1.58	32.44	15.45	2.30	100.19
nodA	psfHH103d_126 / NB8	17.65	-1.59	14.68	30.31	1.49	20.42
-	psfHH103d_161 / NB10	21.58	-1.26	22.37	15.85	2.34	33.05
-	psfHH103d_448 / NB13	27.06	-1.15	28.73	171.06	2.48	144.34
-	psfHH103d_229 / NB14	37.99	-1.21	23.89	39.26	1.59	89.26
y4wE	psfHH103d_257 / NB15	24.69	-1.3	31.5	63.79	1.37	118.87
-	psfHH103d_208 / NB17	17.43	-1.31	39.14	25.93	1.81	86.62
syrM	psfHH103d_367 / NB19	5.81	-1.72	9.29	6.51	1.71	8.47
nopI	SFHH103_04163 / TB2	3.13	-1.22	1.82	24.82	2.39	2.06
gunA	psfHH103d_292 / TB5	5.21	-1.17	2.11	5.49	2.06	1.64
nopL	psfHH103d_327 / TB9	4.56	-2.85	2.31	43.01	1.9	1.84
nopC	psfHH103d_3504 / TB12	6.29	-2.52	1.63	22.52	2.14	2.33
-	psfHH103d_255 / -	3.39	-1.14	3	3.65	1.2	9.51
-	psfHH103d_275 / -	3.45	-1.45	1.42	1.51	-1.2	-1.88
-	psfHH103d_306 / -	20.53	1.21	23.27	103.01	1.3	165.04
flgJ	SFHH103_00346 / -	7.57	-1.3	2.07	5.65	-1.51	-2.38
-	SFHH103_02192 / -	6.9	-1.63	11.64	68.04	-1.23	19.2
-	SFHH103_05321 / -	0.18	0.24	0.17	0.14	0.15	0.1
fbpA	SFHH103_00387	1.03	0.29	0.11	1.07	0.03	0.03

Table S2. 36 and 35 *Sinorhizobium fredii* HH103 ORFs are regulated by genistein through *nod* (NB) and *tts* (TB) boxes respectively[a,b].

**NB/TB**[c]	**Locus-tag (gene name)**	**Functions (putative functions)**	**Fold change**[d]
NB1	psfHH103d_373 to 370	Hypothetical proteins (Carotenoid synthesis)	19.3 to 4.1
NB2	psfHH103d_381 to 379 (*nodZnoeLnolK*)	Fucosylation of LCOs	3.7 to 2.6
NB8	psfHH103d_126 to 132 (*nodABCIJnolO'noeI*)	Synthesis of LCOs backbone / LCO export / Methylation of LCOs	17.7 to 2.4
NB9	psfHH103d_118, 116	Hypothetical proteins	10.3, 4.5
NB10	psfHH103d_161	Hypothetical proteins (Polysaccharide inhibition)	21.6
NB13	psfHH103d_448, 447	Hypothetical proteins (Gene expression regulation?)	27.1, 5.3
NB14	psfHH103d_229 to 224 (*fixABCX*)	Hypothetical proteins/ Electron transfer to nitrogenase	38 to 6.3
NB15	psfHH103d_257 (*y4wE*)	Indole Acetic Acid (IAA) biosynthesis	24.7
NB17	psfHH103d_208	Hypothetical proteins (ABC-type transport)	17.4
NB18	psfHH103d_323 (*ttsI*) to 326	Transcriptional activator of the T3SS / Components of the T3SS apparatus / Conserved putative lipoprotein/(Transposase)	10.1 to 1.5
NB19	psfHH103d_367 (*syrM*) to 369	Transcriptional regulator of symbiotic genes / Hypothetical protein (Transcriptional regulator) / Hypothetical protein (Reduction of ethylene levels)	5.8 to 2.7
TB1	SFHH103_04201 (*nopM2*)	E3 ubiquitinization	2.1
TB2	SFHH103_04163 (*nopI*) to 04167	Putative effector/Hypothetical proteins	3.7 to 3.1
TB3	psfHH103d_90 (*nopM*)	E3 ubiquitinization	2.2
TB4	psfHH103d_287(*nopD*)	(C48 peptidase activity)	7.7
TB5	psfHH103d_292 (*gunA*), 293	Cellulase activity /Hypothetical protein	5.2, 6.5
TB8	SFHH103_335 (*nopX*) to 329	Translocation of T3SS effectors/Hypothetical proteins	10.3 to 3.3
TB9	psfHH103d_327 (*nopL*)	(Host MAPK signalling interference)	4.6

TB10	psfHH103d_337 to 348 (*nopBrhcJnolUrhcLNQRSTU*)	Components of the T3SS apparatus	25.3 to 4.2
TB11	psfHH103d_349 (*nopP*)	(Host MAPK signalling interference)	5.5
TB12	psfHH103d_3504, 3506, 351 to 353 (*nopCAy4yPrhcVy4yS*)	Effector protein/Component of the T3SS apparatus/Component of the T3SS apparatus	7.2 to 4.8
TB13	psfHH103d_363(*nopT*)	C46 peptidase activity	4.5

[a] Genes driven by NB are dependent on NodD1. Genes driven by TB are dependent on both NodD1 and TtsI.
[b] With the exception of genes linked to TB1 and TB2, all these genes are located on the pSym (pl. d, pSfHH103d).
[c] According to Vinardell et al. [20].
[d] Fold induction with respect to non-induced cultures. In operons, the first and second values correspond to the first and last gene of the transcriptional unit respectively.

Table S3. 30 *Sinorhizobium fredii* HH103 ORFs are regulated by genistein but are not dependent on *nod* or *tts* boxes.

**17 genes depending on NodD1 (12/5)**[a]	**Induced**:<br>SFHH103_00841(*ligE*), SFHH103_02192, SFHH103_06013, psfHH103d_255, psfHH103d_306 to 311, psfHH103d_322 to 319 (including *nodD2*)<br>**Repressed**:<br>SFHH103_00450, SFHH103_00451, SFHH103_00799, SFHH103_01317, SFHH103_02323 (*hmuU*)
**7 genes depending on NodD1 and TtsI (7/0)**	**Induced:**<br>SFHH103_00346 (*flgJ*) to 00348, SFHH103_00844, SFHH103_01920, psfHH103d_275 and psfHH103d_274
**6 genes no depending on NodD1 and/or TtsI (3/3)**	**Induced**:<br>SFHH103_03274[b], SFHH103_03749[c], SFHH103_03875<br>**Repressed**:<br>SFHH103_05321 to 05319

[a] The number of genes induced/repressed is shown between brackets.
[b] The induction of this genes was 3.0, 2.4, and 2.7 in the wt, *nodD1* and *ttsI* backgrounds respectively.
[c] This gene only shows partial induction in the *ttsI* mutant background

Supplementary Data 1. General features of the total sequenced and mapped reads.

Total Number of Reads: Reads were mapped and pair using a Bayesian inference using Cufflinks v2.11 software. Worst quality reads were removed by means of Picard Tools.

**Sample condition**	**#Mapped Reads**	**High Quality Mapped Reads**	**Properly Pair**
Wild-type_1	43,514,666	41,856,055	40,447,758
Wild-type_2	47,976,366	46,001,800	43,889,282
Wild-type_GEN_1	41,545,412	39,919,415	38,189,900
Wild-type_GEN_2	44,649,058	43,054,550	41,288,654
NodD1$^-$_1	42,853,472	40,660,931	38,861,148
NodD1$^-$_2	39,211,312	37,460,139	35,672,278
NodD1$^-$_GEN_1	43,075,850	41,447,019	39,962,702
NodD1$^-$_GEN_2	44,443,662	42,776,374	40,794,302
TtsI$^-$_1	46,494,272	44,688,429	43,212,764
TtsI$^-$_2	46,671,594	38,746,887	35,760,244
TtsI$^-$_GEN_1	41,472,444	39,520,075	37,767,084
TtsI$^-$_GEN_2	45,623,306	13,074,049	12,332,926

Two biological and independent experiments were carried out for each condition.

Quality Read Controls: Three different controls were performed to ensure the quality of reads: GC content, duplicate distribution and distribution respect genetic coordinates.

- *GC content*: distribution of GC content on mapped reads. A normal distribution around 45-55% is expected.

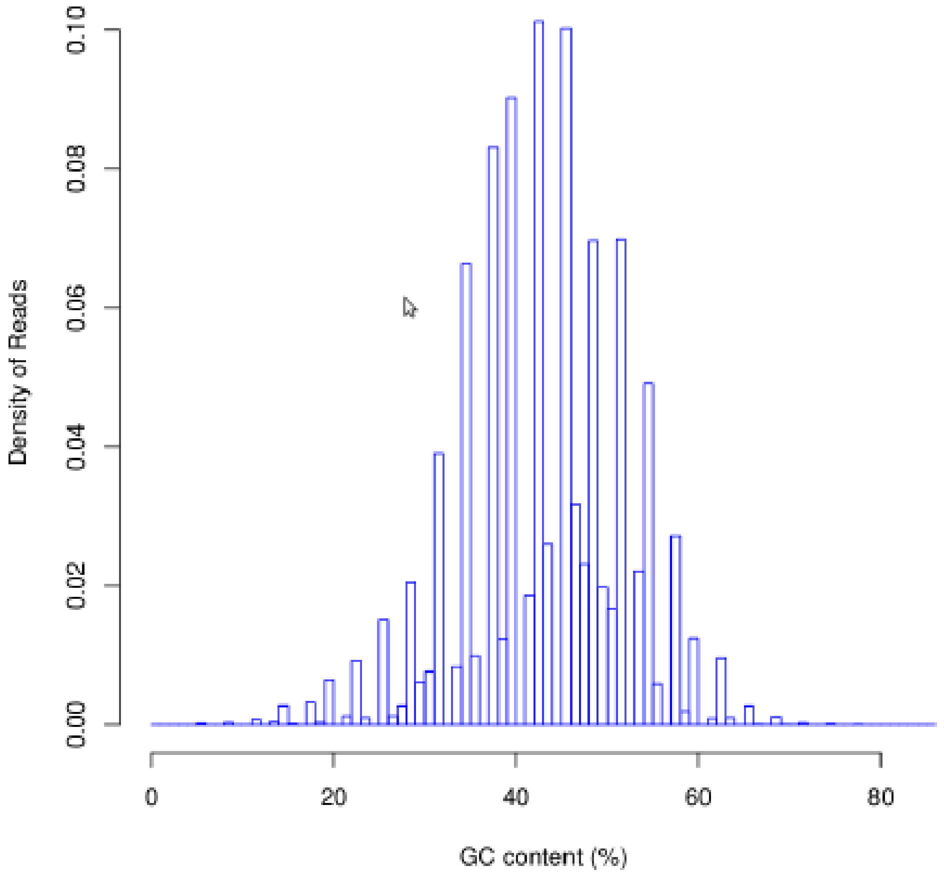

- *Duplicate distribution*: common distribution of duplicates in a RNA-Seq experiment shows a small number of reads with high levels of duplicates and a high number of reads with low levels of duplicates. All samples presented optimal values of duplicate distributions.

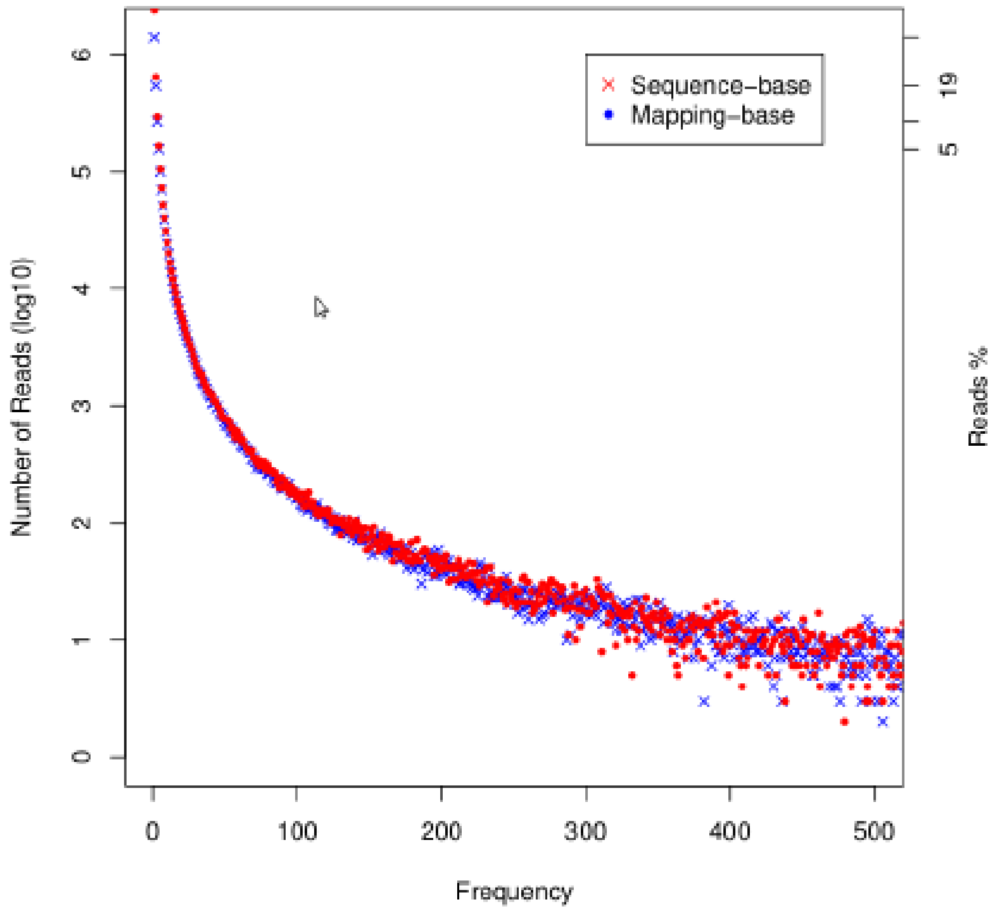

- *Distribution respect genetic coordinates*: centralization around 50 percentile of gene body is expected in high quality samples. Read concentration in both ends could be indicative of RNA degradation.

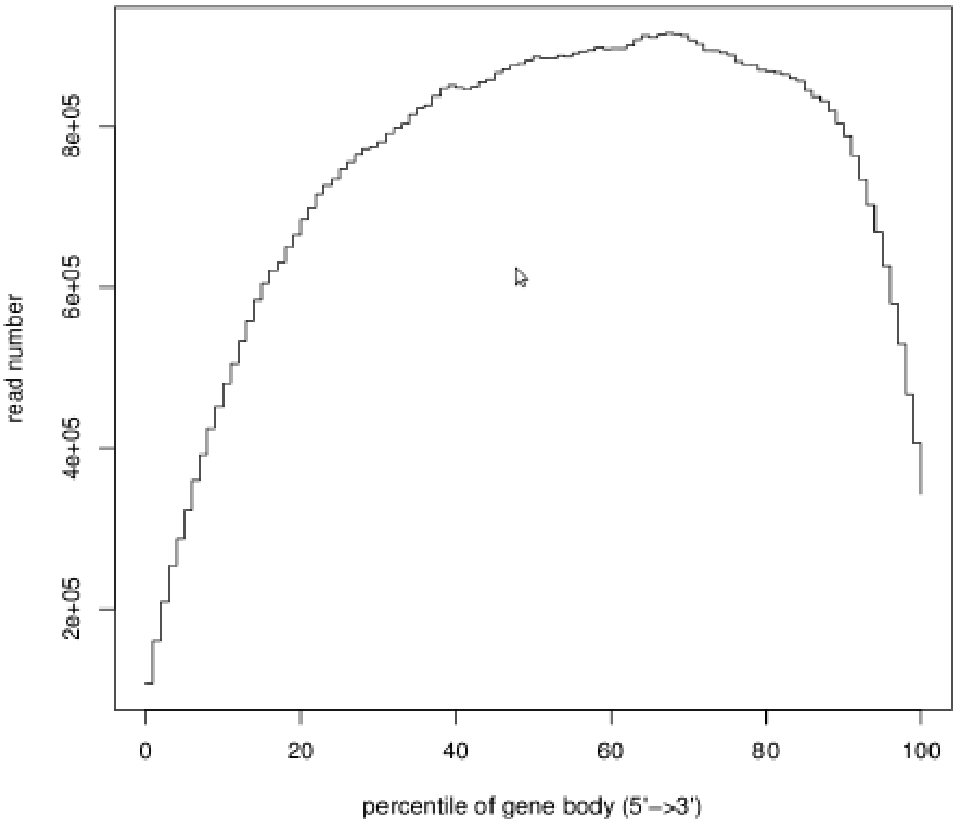

Normalization: normalization is needed to avoid statistical deviations due to differences in library sizes.

Number of reads per gene **before** normalization.

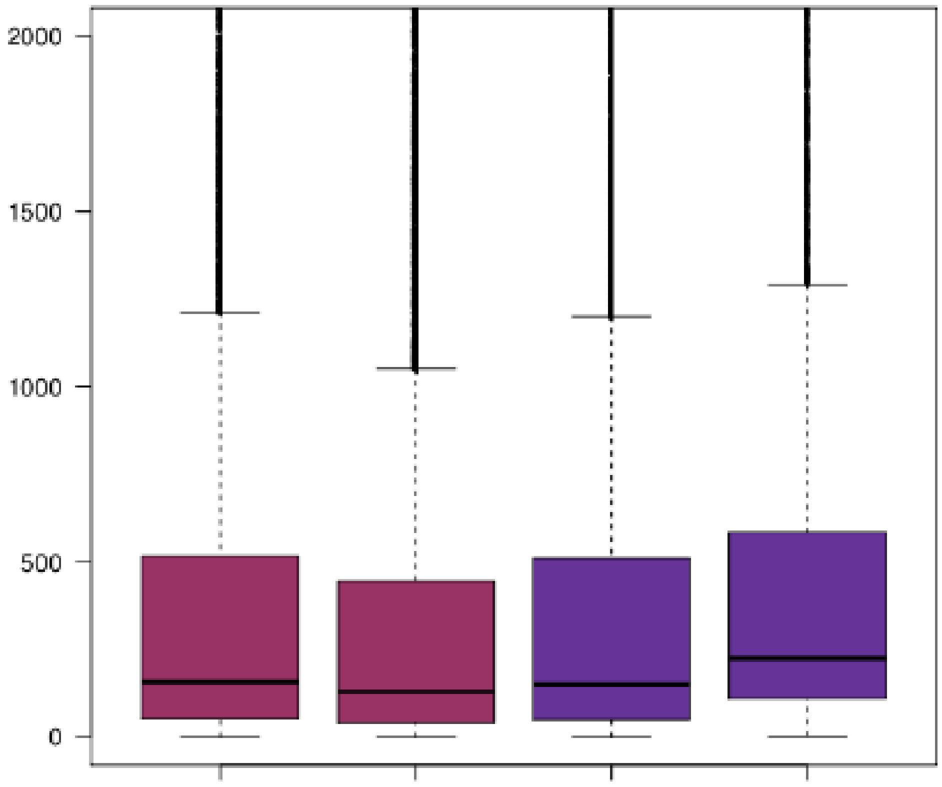

1 **2** **1** **2**

Wild-type_GEN **Wild-type**

Number of reads per gene **before** normalization.

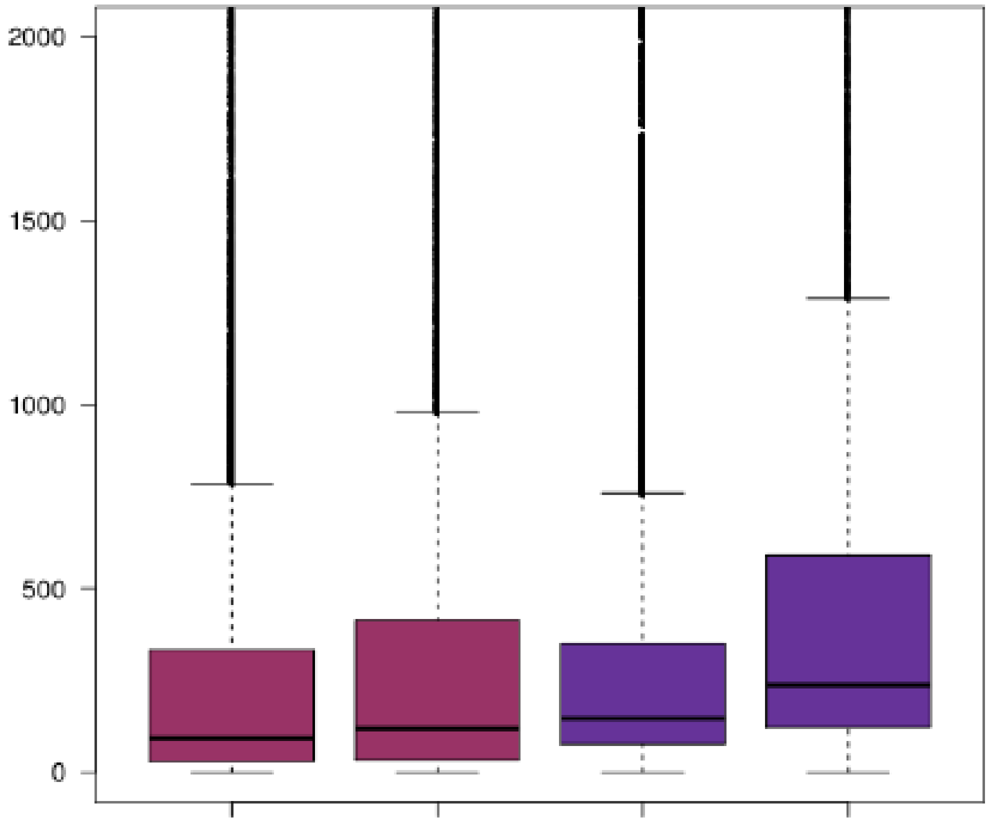

1 **2** **1** **2**

NodD1^{-}_GEN **NodD1^{-}**

Number of reads per gene **before** normalization.

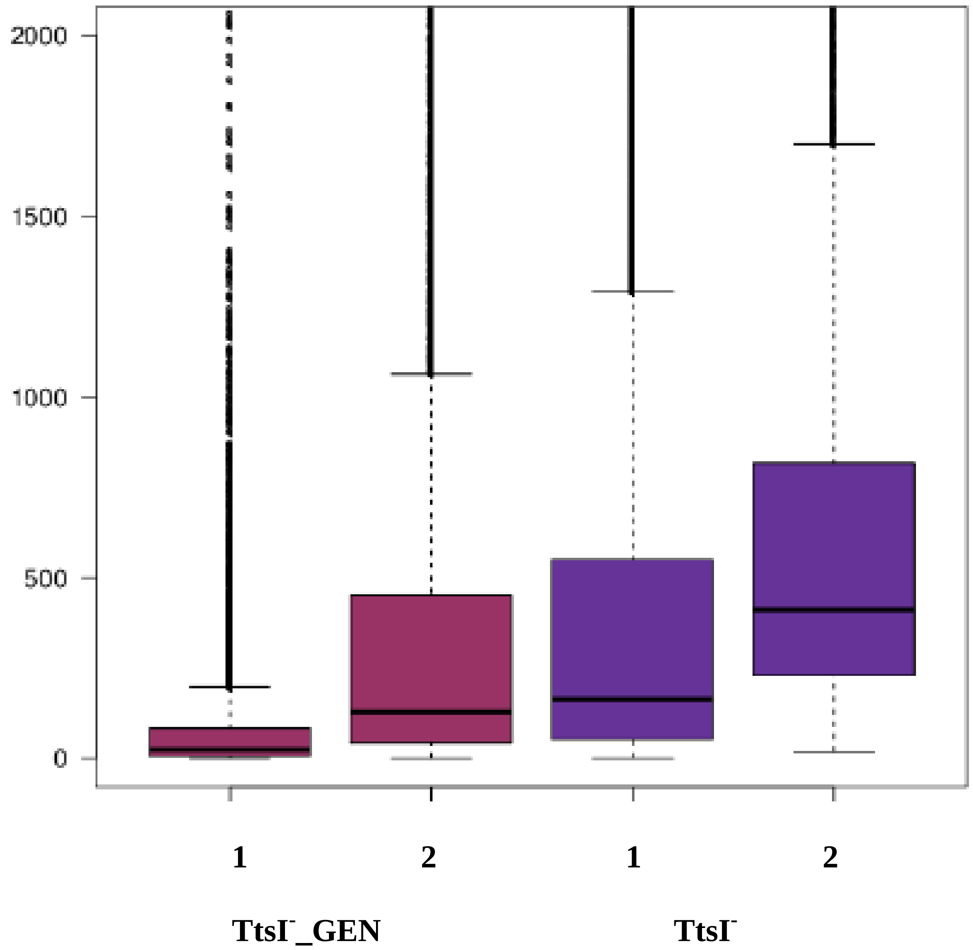

Number of reads per gene **after** normalization.

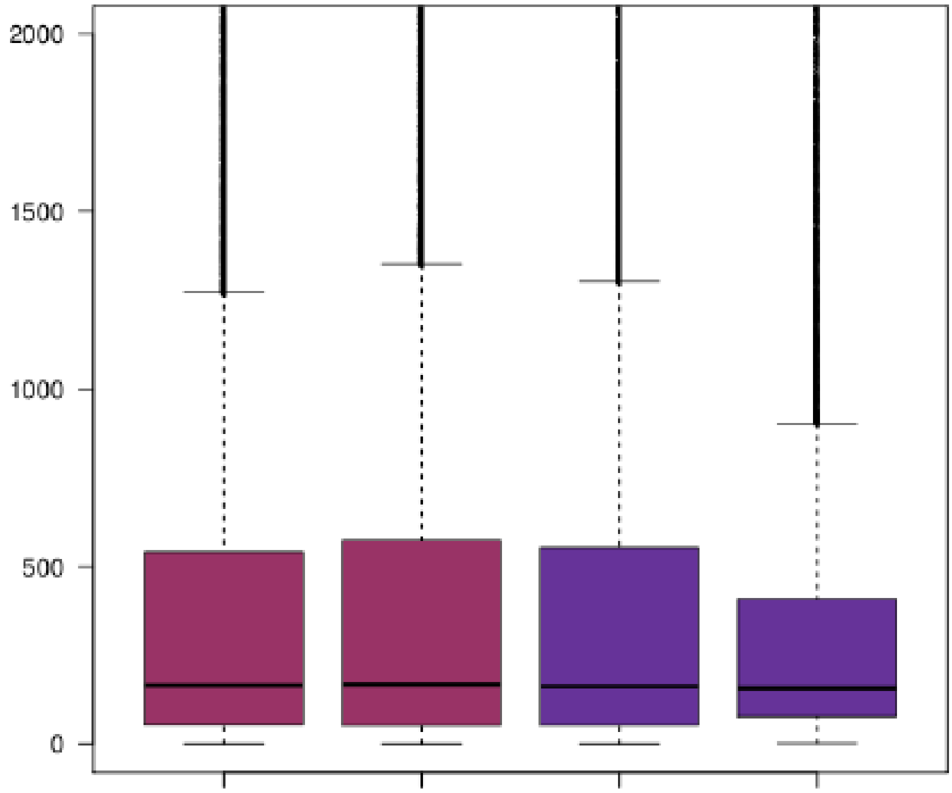

1 **2** **1** **2**

Wild-type_GEN **Wild-type**

Number of reads per gene **after** normalization.

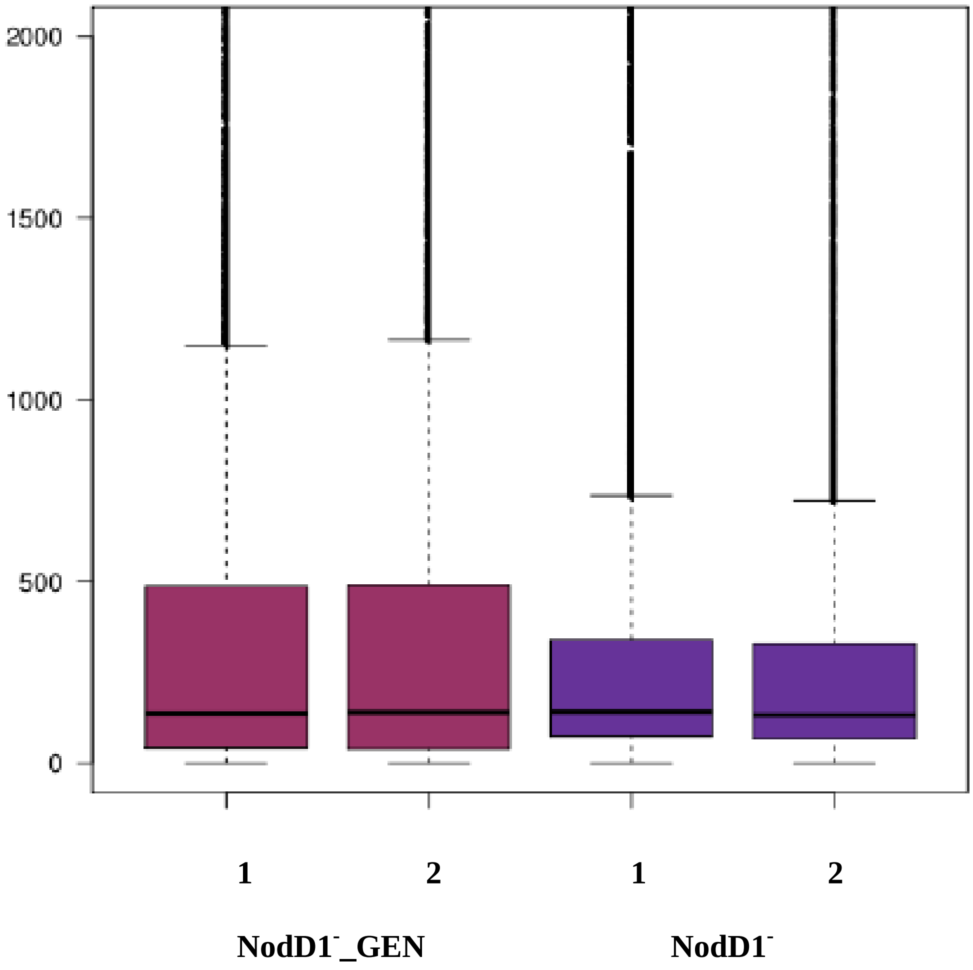

Number of reads per gene **after** normalization.

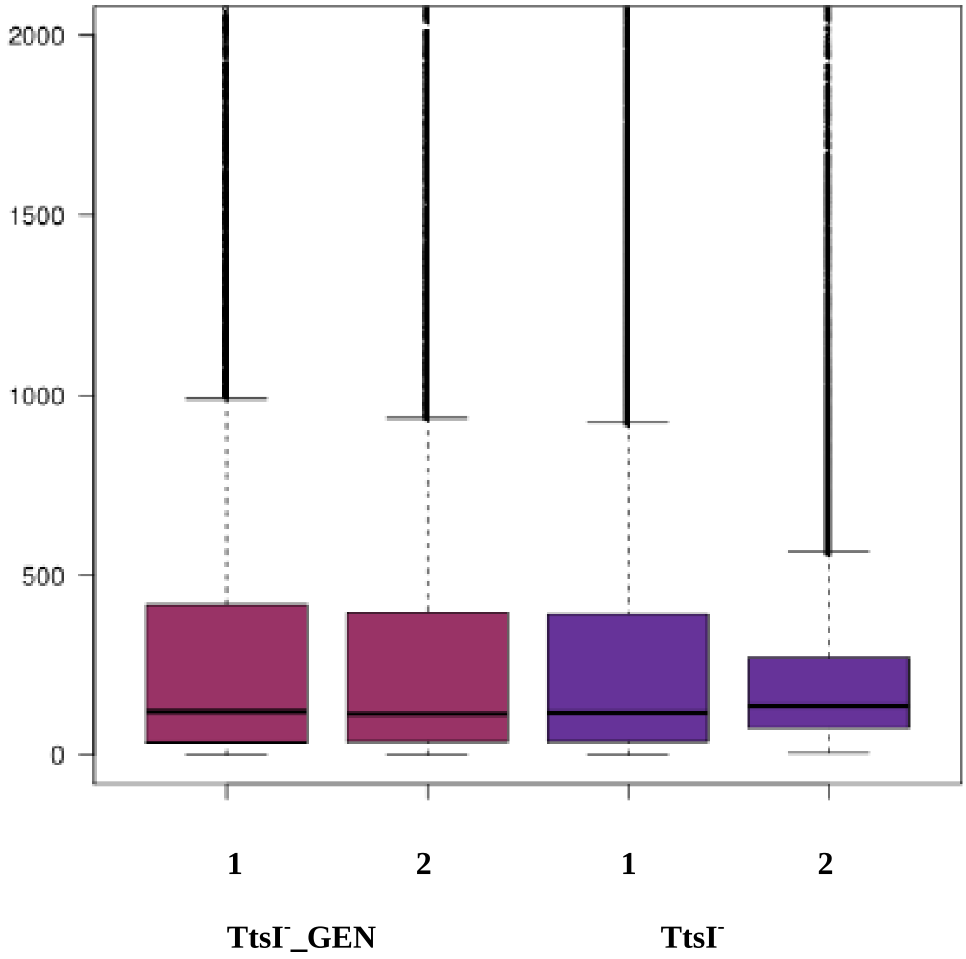

References cited in Supplementary Data 4

Ausmees, N. et al. Characterization of NopP, a type III secreted effector of *Rhizobium* sp. strain NGR234. J. Bacteriol. **186,** 4774-4780 (2004).

Bartsev, A. V., Boukli, N. M., Deakin, W. J., Staehelin, C. & Broughton, W. J. Purification and phosphorylation of the effector protein NopL from *Rhizobium* sp. NGR234. FEBS Lett. **554,** 271-274 (2003).

Bartsev, A. V. et al. NopL, an effector protein of *Rhizobium* sp. NGR234, thwarts activation of plant defense reactions. Plant Physiol. **134,** 871-879 (2004).

Bellato, C., Krishnan, H. B., Cubo, T., Temprano, F. & Pueppke, S. G. The soybean cultivar specificity gene *nolX* is present, expressed in a *nodD*-dependent manner, and of symbiotic significance in cultivar-nonspecific strains of *Rhizobium* (*Sinorhizobium*) *fredii*. Microbiology. **143,** 1381-1388 (1997).

Caldelari Baumberger, I., Fraefel, N., Göttfert, M. & Hennecke, H. New NodW- or NifA-regulated *Bradyrhizobium japonicum* genes. Mol. Plant Microbe Interact. **16,** 342-351 (2003).

Dai, W. J., Zeng, Y., Xie, Z. P. & Staehelin, C. Symbiosis-promoting and deleterious effects of NopT, a novel type 3 effector of *Rhizobium* sp. strain NGR234. J. Bacteriol. **190,** 5101-110 (2008).

de Lyra, Mdo C. et al. Inactivation of the *Sinorhizobium fredii* HH103 *rhcJ* gene abolishes nodulation outer proteins (Nops) secretion and decreases the symbiotic capacity with soybean. Int. Microbiol. **9,** 125-133 (2006).

Deakin, W. J., Marie, C., Saad, M. M., Krishnan, H. B. & Brougthon, W. J. NopA is associated with cell surface appendages produced by the type III secretion system of *Rhizobium* sp. strain NGR234. Mol. Plant Microbe Interact. **18,** 499-507 (2005).

Earl, C. D., Ronson, C. W. & Ausubel, F. M. Genetic and structural analysis of the *Rhizobium meliloti fixA*, *fixB*, *fixC*, and *fixX* genes. J. Bacteriol. **169,** 1127-1136 (1987).

Hubber, A., Vergunst, A. C., Sullivan, J. T., Hooykaas, P. J. & Ronson, C. W. Symbiotic phenotypes and translocated effector proteins of the *Mesorhizobium loti* strain R7A VirB/D4 type IV secretion system. Mol Microbiol. **54,** 561-574 (2004).

Jabbouri, S, et al. *nolO* and *noeI* (HsnIII) of *Rhizobium* sp. NGR234 are involved in 3-O-carbamoylation and 2-O-methylation of Nod factors. J. Biol. Chem. **273,** 12047-12055 (1988).

Jiang, G. & Krishnan, H. B. *Sinorhizobium* fredii USDA257, a cultivar-specific soybean symbiont, carries two copies of *y4yA* and *y4yB*, two open reading frames that are located in a region that encodes the type III protein secretion system. Mol. Plant Microbe Interact. **13,** 1010-1014 (2000).

Jiménez-Guerrero, I., Pérez-Montaño, F., Medina, C., Ollero, F. J. & López-Baena, F. J. NopC is a *Rhizobium*-specific type 3 secretion system effector secreted by *Sinorhizobium (Ensifer) fredii* HH103. PLoS One **10,** e0142866 (2015).

Kambara, K. et al. Rhizobia utilize pathogen-like effector proteins during symbiosis. Mol. Microbiol. **71,** 92-106 (2009).

Kim, W. S. & Krishnan, H. B. A *nopA* deletion mutant of *Sinorhizobium fredii* USDA257, a soybean symbiont, is impaired in nodulation. Curr. Microbiol. **68,** 239-246 (2014).

Kondorosi, E. et al. Involvement of the *syrM* and *nodD3* genes of *Rhizobium meliloti* in *nod* gene activation and in optimal nodulation of the plant host. Mol Microbiol. **5,** 3035-3048 (1991).

Kovács, L. G., Balatti, P. A., Krishnan, H. B. & Pueppke, S. G. Transcriptional organization and expression of *noIXWBTUV*, a locus that regulates cultivar-specific nodulation of soybean by *Rhizobium fredii* USDA257. Mol Microbiol. **17,** 923-933 (1995).

Krishnan, H. B. & Pueppke, S. G. Flavonoid inducers of nodulation genes stimulate *Rhizobium fredii* USDA257 to export proteins into the environment. Mol. Plant Microbe Interact. **6,** 107-113 (1993).

Krishnan, H. B. et al. Extracellular proteins involved in soybean cultivar-specific nodulation are associated with pilus-like surface appendages and exported by a type III protein secretion system in *Sinorhizobium fredii* USDA257. Mol. Plant Microbe Interact. 2003 **16,** 617-625 (2003).

Kulkarni, G. et al. Specific hopanoid classes differentially affect free-living and symbiotic states of *Bradyrhizobium diazoefficiens*. MBio. **6,** 5 (2015).

Lamrabet, Y. et al. Mutation in GDP-fucose synthesis genes of *Sinorhizobium fredii* alters Nod factors and significantly decreases competitiveness to nodulate soybeans. Mol. Plant Microbe Interact. **12,** 207-217 (1999).

López-Baena, F. J. et al. Regulation and symbiotic significance of nodulation outer proteins secretion in *Sinorhizobium fredii* HH103. Microbiology **154,** 1825-1836 (2008).

López-Baena, F. J. et al. The absence of Nops secretion in *Sinorhizobium fredii* HH103 increases GmPR1 expression in Williams soybean. Mol. Plant Microbe Interact. **22,** 1445-1454 (2009).

Lorio, J. C., Kim, W. S. & Krishnan, H. B. NopB, a soybean cultivar-specificity protein from *Sinorhizobium fredii* USDA257, is a type III secreted protein. Mol. Plant Microbe Interact. **17,** 1259-1268 (2004).

Lorio, J. C., Chronis, D. & Krishnan, H. B. Y4xP, an open reading frame located in a type III protein secretion system locus of *Sinorhizobium fredii* USDA257 and USDA191, encodes cysteine synthase. Mol. Plant Microbe Interact. **19,** 635-643 (2006).

Madinabeitia, N. et al. *Sinorhizobium fredii* HH103 has a truncated *nolO* gene due to a -1 frameshift mutation that is conserved among other geographically distant *S. fredii* strains. Mol. Plant-Microbe Interact. **15,** 150-159 (2002).

Marie, C. et al. Characterization of Nops, nodulation outer proteins, secreted via the type III secretion system of NGR234. Mol. Plant Microbe Interact. **16,** 743-751 (2003).

Meinhardt, L. W., Krishnan, H. B., Balatti, P. A. & Pueppke, S. G. Molecular cloning and characterization of a sym plasmid locus that regulates cultivar-specific nodulation of soybean by *Rhizobium fredii* USDA257. Mol Microbiol. **9,** 17-29 (1993).

Mercante, V. et al. The absence of protein Y4yS affects negatively the abundance of T3SS *Mesorhizobium loti* secretin, RhcC2, in bacterial membranes. Front. Plant Sci. **6,** 12 (2015).

Miller, S.H. , Elliot, R. M., Sullivan, J. T. & Ronson, C. W. Host-specific regulation of symbiotic nitrogen fixation in *Rhizobium leguminosarum* biovar *trifolii*. Microbiology. **153,** 3184-3195 (2007).

Okazaki, S., Kaneko, T., Sato, S. & Saeki, K. Hijacking of leguminous nodulation signaling by the rhizobial type III secretion system. Proc. Natl. Acad. Sci. USA. **110,** 17131-17136 (2013).

Oldroyd, G. E. Speak, friend, and enter: signaling systems that promote beneficial symbiotic associations in plants. Nat. Rev. Microbiol. **11,** 252-263 (2013).

Silipo, A. et al. Covalently linked hopanoid-lipid A improves outer-membrane resistance of a *Bradyrhizobium* symbiont of legumes. Nat. Commun. **5,** 5106 (2014).

Skorpil, P. et al. NopP, a phosphorylated effector of *Rhizobium* sp. strain NGR234, is a major determinant of nodulation of the tropical legumes *Flemingia congesta* and *Tephrosia vogelii*. Mol. Microbiol. **57,** 1304-1317 (2005).

Staehelin, C. & Krishnan, H. B. Nodulation outer proteins: double-edged swords of symbiotic rhizobia. Biochem. J. **470,** 263-274 (2015).

Theunis, M., Kobayashi, H., Broughton, W. J. & Prinsen, E. Flavonoids, NodD1, NodD2, and *nod*-box NB15 modulate expression of the *y4wEFG* locus that is required for Indole-3-Acetic Acid Synthesis in *Rhizobium* sp. strain NGR234. Mol. Plant Microbe Interact. **17,** 1153-1161 (2004).

Tittabutr, P., Awaya, J. D., Li, Q. X. & Borthakur, D. The cloned 1-aminocyclopropane-1-carboxylate (ACC) deaminase gene from *Sinorhizobium* sp. strain BL3 in *Rhizobium* sp. strain TAL1145 promotes nodulation and growth of *Leucaena leucocephala*. Syst. Appl. Microbiol. **31,** 141-150 (2008).

Tsurumaru, H. et al. A Putative Type III Secretion System Effector Encoded by the MA20_12780 Gene in *Bradyrhizobium japonicum* Is-34 Causes Incompatibility with Rj4 Genotype Soybeans. Appl. Environ. Microbiol. **81,** 5812-5819 (2015).

van Rhijn, P. & Vanderleyden, J. The *Rhizobium*-plant symbiosis. Microbiol. Rev. **59,** 124-142 (1995).

Viprey, V., Del Greco, A., Golinowski, W., Broughton, W. J. & Perret, X. Symbiotic implications of type III protein secretion machinery in *Rhizobium*. Mol. Microbiol. **28,** 1381-1389 (1998).

Xin, D. W. et al. Functional analysis of NopM, a novel E3 ubiquitin ligase (NEL) domain effector of *Rhizobium* sp. strain NGR234. PLoS Pathog. **8,** e1002707 (2012).

Zhang, L., Chen, X. J., Lu, H. B., Xie, Z. P. & Staehelin, C. Functional analysis of the type 3 effector nodulation outer protein L (NopL) from *Rhizobium* sp. NGR234: symbiotic effects, phosphorylation, and interference with mitogen-activated protein kinase signaling. J. Biol. Chem. 2011 **286,** 32178-32187 (2011).
